# Evaluating Reporting Completeness in Oral Health Clinical Guidelines: A Meta-Research Study

**DOI:** 10.1177/23800844251357235

**Published:** 2025-08-16

**Authors:** P. Lancry, P. Lyra, J.J. Mendes, G.G. Nascimento, V. Machado, J. Botelho

**Affiliations:** 1 Egas Moniz Center for Interdisciplinary Research, Egas Moniz School of Health and Science, Almada, Portugal; 2 National Dental Research Institute Singapore, National Dental Centre Singapore, Singapore; 3 Oral Health Academic Programme, Duke-NUS Medical School, Singapore

**Keywords:** evidence-based medicine, guidelines, reporting guidelines, agree, oral health, meta-research

## Abstract

**Background:**

To assess the adherence to clinical practice guidelines (CPGs) reporting guidelines for oral health.

**Methods:**

A literature search was carried out in PubMed, Embase and Web of Science from March 2016 to December 2023, selecting CPGs related to oral health. The study selection and data extraction were conducted independently by 2 researchers. Guidelines were cross-checked against the 23-item Appraisal of Guidelines, Research, and Evaluation (AGREE). The results were then collected, and the overall adherence and adherence to each AGREE item and section were calculated. Regression analyses were performed considering journal characteristics, such as quartile, year, and publishing options in journals’ guideline endorsement. There were no language restrictions.

**Results:**

Twenty-one CPGs were included in this study. The mean overall AGREE adherence was 48.7%. The results showed considerable variability in the rates of compliance with the reporting guidelines. Three areas appear to have (much) higher levels of compliance than the others, notably “Clarity of Presentation” (83.6%, 95% confidence interval [CI]: 75.4%–91.8%), “Scope and Purpose” (74.0%, 95% CI: 67.1%–80.9%), and “Stakeholder Involvement” (54.5%, 95% CI: 43.4%–65.7%). The lowest level of agreement was found in “Applicability,” with a level of agreement of 18.5% (95% CI: 11.4%–25.6%). Four of the 6 domains had a complete lack (0.0%) of adherence. Journal quartiles were significant, as guidelines published in the second- (B = −27.3%; standard error [SE] = 6.1) and third-quartile (B= −22.8%; SE = 10.6) impact factor journals displayed a lower overall adherence than those published in first-quartile journals. Guideline endorsement by journals was also a significant variable (B = −20.9%, SE = 5.9).

**Conclusion:**

Reporting completeness in dental/oral CPGs is suboptimal and is associated with the journal’s quartile and guideline endorsement. Increasing awareness of CPG reporting guidelines and ensuring their rigorous application are decisive toward better adherence.

**Knowledge Transfer Statement:**

The quality of reporting in dental and oral clinical practice guidelines (CPGs) is low and linked to the journal’s impact factor and the endorsement of the guidelines. Enhancing knowledge of CPG reporting guidelines and implementing them rigorously are critical for improved adherence.

## Introduction

Oral health research plays a pivotal role in advancing the understanding of dental diseases, treatment modalities, and preventive strategies (Peres et al. 2019; Watt et al. 2019; Botelho et al. 2022). The translation of research findings into clinical practice relies heavily on the accurate and transparent reporting of study methods and results (Institute of Medicine Committee on Standards for Developing Trustworthy Clinical Practice Guidelines et al. 2011). As such, clinical practice guidelines (CPGs) provide standardized frameworks to ensure comprehensive and transparent reporting of research studies (AGREE Collaboration 2003; Brouwers et al. 2010; Institute of Medicine Committee on Standards for Developing Trustworthy Clinical Practice Guidelines et al. 2011). Compliance with these guidelines is essential for improving the dependability, replicability, and applicability of research outcomes in the realm of dental health.

The suboptimal adherence to reporting guidelines is unfortunately prevalent across several disciplines, and oral health is no exception. For example, a meta-research study on exposure to organic pollutants demonstrated a low rate of compliance with the analyzed guidelines, with only 47.0% following the Strengthening the reporting of observational studies in epidemiology (STROBE) guidelines and 43.1% adhering to the SEMinal QUAlity studies (SEMQUA) guidelines (Serrano et al. 2014). Similarly, oncology research has shown poor reporting of the methodological aspects of randomized trials, with only 10.0% and 15.0% adherence to Consolidated Standards of Reporting Trials (CONSORT) standards for collection and analysis, respectively (Péron et al. 2013).

Nonadherence to reporting guidelines can result in biased or incomplete reporting, which hinders the synthesis, interpretation, and translation of research findings by clinicians, policymakers, and other stakeholders (Simera and Altman 2009). In addition, inadequate reporting may undermine the credibility and trustworthiness of research publications, ultimately affecting patient care and clinical decision-making (Dickersin and Chalmers 2011). In the context of dental and oral health, Sarkis-Onofre et al. (2020) reported that the endorsement of CONSORT improved the reporting quality of randomized clinical trials in dentistry. There is also evidence indicating suboptimal adherence to guidelines in oral health research until 2014 (Sarkis-Onofre et al. 2016; Mubeen et al. 2017), yet little is known later on.

Therefore, it is imperative to evaluate the extent of adherence to clinical practice reporting guidelines in oral health research to identify areas for improvement to promote transparency and rigor in the field and, ultimately, improve the quality of dental research. In response to this need, the present meta-research study was designed to systematically assess adherence to CPG reporting guidelines in published oral health research. Ultimately, the findings of this study have the potential to inform strategies for improving reporting practices, enhancing the credibility and utility of oral health research and contributing to improved patient outcomes and CPGs.

## Materials and Methods

This meta-research study aimed to evaluate reporting completeness in oral health CPGs. The research question was, “What is the percentage of adherence and completeness of oral health CPGs for each item and domain of the AGREE reporting guideline?” The study also aimed to explore further confounding variables. This study is reported following the guideline for reporting meta-epidemiological methodology research by Murad and Wang (2017) (Appendix Table S1).

### Study Selection Criteria and Search Strategy

A cross-sectional analysis was conducted for a consecutive sample of CPGs related to oral health fields of expertise, including cariology, periodontology, oral surgery, implantology, and endodontics, among others. The search strategy sought to identify CPGs that were conducted between March 2016 and December 2023. CPGs from 2016 onward were selected considering the year of publication of the Appraisal of Guidelines, Research, and Evaluation (AGREE) Reporting Checklist (Brouwers et al. 2016). There were no restrictions on language or country of publication. In line with this, potentially eligible CPGs in oral health published between March 2016 and December 2023 as full-text scientific articles were considered.

To be included, CPGs had to (1) have been submitted in the search period defined, (2) have reference to CPG in the title (or synonym), (3) be dedicated to oral health research, and (4) have access to the methodology. If not respecting these inclusion criteria, studies were excluded. Also, articles with an abstract only, in vitro studies, animal studies, case reports or case series, cross-sectional studies, patient handouts, narrative reviews, mixed-methods reviews, qualitative evidence synthesis, umbrella reviews, scoping reviews, editorials, letters to the editor, and news reports were also excluded.

The following search syntax was used to search CPGs in PubMed and adapted to other search databases (Embase and Web of Science) (detailed as Appendix Table 2).

### Study Selection Process

Two researchers (P.L. and J.B.) independently selected articles by screening titles and abstracts and excluding nonrelevant studies. Any articles deemed potentially eligible by either reviewer were assessed in their entirety, with the reasons for exclusion being thoroughly documented (Appendix Table 3). In cases in which a disagreement occurred, a third reviewer (V.M.) was consulted, and a consensus decision was achieved.

### Data Extraction

Descriptive data were obtained from the included studies, including first author, publication year, publishing organization, theme, funding information, publishing option, and journal impact factor ranking via the Journal Citation Reports Clarivate (https://jcr.clarivate.com/jcr/browse-journals). Journal quartiles were used as a proxy of impact factors within its field or research. For the publication option (open access or hybrid), we explored the article status. Two reviewers performed data extraction independently (P.L. and J.B.), and any disagreement was resolved by a third reviewer (V.M.).

### Assessing the Completeness of Reporting

The completeness of reporting was calculated as adherence to the 23-item AGREE checklist (Brouwers et al. 2016). Potential discrepancies between authors on the application of elements of the AGREE checklist were resolved by consensus. Interrater agreement was assessed for 4 guidelines (15% of the entire sample), which were independently rated by 2 authors (P.L. and J.B.) with postgraduate training in clinical epidemiology and critical appraisal. The detailed analysis was conducted using The AGREE Reporting Checklist (PDF version) available on the official EquatorNetwork website (https://www.equator-network.org/).

Following the explanations and developments of the AGREE checklist, each element was marked with a “1” if it was as per the AGREE checklist description or a “0” if not.

Adherence to each item and overall adherence to the AGREE checklist for each study were calculated (from 0% to 100%, with 0 representing zero adherence and 100% total adherence). In this way, each item represented in the different elements has its own adherence score. As the AGREE checklist is organized into 23 items divided into 6 main domains, statistics were calculated for each of the 23 items and each of the 6 main domains.

The total completion rate was also assessed by weighting the number of studies that completed all the items in each of the 23 items by the total number of guidelines analyzed. This completion rate was also calculated for each item and for each of the 6 domains of the AGREE checklist.

As the aim of this study was to assess adherence to clinical reporting guidelines, the authors of the included studies were not contacted to obtain information omitted from the manuscripts.

To assess the reliability of the examiners’ evaluations, both were trained and then calibrated using 5 CPGs, which were unrelated to the final sample. The 2 evaluators independently reviewed and scored these items. Of the 115 total items, the evaluators agreed on 107 items and disagreed on 8 items. The interrater agreement was 93.0% (95% confidence interval [CI]: 0.884, 0.977).

To aggregate the scores provided by the 2 independent evaluators, a systematic approach was followed. Each guideline was first reviewed separately by both evaluators, who assigned their scores based on the AGREE checklist. Once the individual evaluations were completed, the scores were compared and combined. As the final score is binomial (yes vs. no), when the reviewers disagreed, a consensus approach was employed using a third examiner (V.M.). This involved a discussion to resolve differences, with the goal of achieving a more balanced and representative final score. This method ensured both rigor and fairness in the aggregation process, reducing bias and improving the reliability of the final assessment. Thus, the analyses comprised the consensus between both reviewers’ scores.

### Statistical Analysis

Adherence to the different criteria in each study (see above) was calculated as the overall adherence to the AGREE checklist. This was estimated (as a percentage) as the total number of items described and related to the total number of applicable items.

Total completion in each of the 23 items and each of the 6 domains was calculated as the total adherence (i.e., 100%) to 1 of the 23 items or 1 of the 6 main domains included in the AGREE checklist. This rate was also estimated (as a percentage) as the number of guidelines that meet all the criteria (of the group or main domain), divided by the total number of applicable elements.

Inferential statistics, mean and 95% CIs, and linear regression analyses were performed using R to assess the association between the overall and item-wise completeness (calculated as the overall adherence to the AGREE reporting checklist) as the dependent variable and the following characteristics as independent variables: publication year, journal ranking (quartile range: Q1, Q2, Q3, Q4), publication options (open access vs. hybrid), and reporting guideline endorsement by the journal during submission. The analysis began with a crude multivariable regression, and the significant variables (*P* < 0.05) were included in an adjusted regression analysis offering a more comprehensive and controlled approach by accounting for relevant covariates to better understand the true association between variables.

## Results

### Characteristics of the Included Guidelines

From a total of 10,463 entries, and after excluding duplicates, 32 articles were obtained for full-paper appraisal. After reading and analyzing the included studies, 11 were excluded (the list and respective reasons for exclusion are detailed in Appendix Table 3 and Appendix Figure 1, respectively). The final list, therefore, contains 21 guidelines to be analyzed.

To ensure the transparency of the results, they have all been reported in a summary table of the 23 items divided into 6 different domains (raw data available for download in the Data Availability Statement).

Of the 21 guidelines selected, only 4 (19.0%) fell into the pharmacology category and 2 (9.5%) into the diagnosis category, and the prevention/handling theme corresponded to 5 (23.8%) of the studies selected (Table 1). Most studies, 15 (71.4%) guidelines, were directly related to the treatment itself. The publication dates of the guidelines covered by this study range from August 2016 to September 2023, for the most recent.

**Table 1.** Characteristics of the Included Guidelines.

Study (Year)	Source (Q)	Field	Reporting Guideline	Funding	Publishing Option	Guideline Endorsement by the Journal
Carrasco-Labra et al. (2023)	Journal of the ADA (Q1)	Dental pharmacology	NR	Industry, research, and institutional funds disclosed	Open access	Yes
Dhar et al. (2023)	Journal of the ADA (Q1)	Cariology	AGREE	Industry, research, and institutional funds disclosed	Open access	Yes
Herrera et al. (2023)	Journal of Clinical Periodontology (Q1)	Implantology	AGREE	EFP internal funds	Open access	Yes
Pietropaoli et al. (2023)	High Blood Pressure and Cardiovascular Prevention (Q2)	Periodontology	NR	None	Open access	Yes
Herrera et al. (2022)	Journal of Clinical Periodontology (Q1)	Periodontology	AGREE	EFP internal funds	Open access	Yes
Sanz et al. (2022)	Journal of Clinical Periodontology (Q1)	Periodontology	AGREE	EFP internal funds	Open access	Yes
Bourguignon et al. (2020)	Dental traumatology (Q2)	Dental traumatology	NR	None	Open access	No
Fouad et al. (2020)	Dental traumatology (Q2)	Dental traumatology	NR	NR	Open access	No
Day et al. (2020)	Dental traumatology (Q2)	Dental traumatology	NR	NR	Open access	No
Farooqi et al. (2020)	International Journal of Oral and Maxillofacial Surgery (Q2)	Dental pharmacology	NR	None	Open access	No
Yarom et al. (2020)	Journal of Clinical Oncology (Q1)	Dental pharmacology and oral pathology	NR	Industry, research, and institutional funds disclosed	Open access	No
Alharbi et al. (2020)	Saudi Dental Journal (Q2)	COVID-19	NR	NR	Open access	No
Lockhart et al. (2019)	Journal of the ADA (Q1)	Oral pathology	AGREE	Industry, research, and institutional funds disclosed	Open access	Yes
Ricucci et al. (2019)	Journal of Dentistry (Q1)	Endodontics	NR	NR	Open access	No
Toumba et al. (2019)	European Archives of Paediatric Dentistry (Q3)	Cariology	NR	NR	Open access	Yes
Slayton et al. (2018)	Journal of the ADA (Q1)	Cariology	AGREE	Industry, research, and institutional funds disclosed	Open access	Yes
Lingen et al. (2017)	Journal of the ADA (Q1)	Oral pathology	AGREE	Industry, research, and institutional funds disclosed	Open access	Yes
Dhar et al. (2017)	Pediatric Dentistry (NA)	Endodontics	AGREE	The authors wish to thank the AAP for their financial and administrative support	Open access	NA
Crystal et al. (2017)	Pediatric Dentistry (NA)	Cariology	AGREE	The preparation of this guideline was funded by the AAPD	Open access	NA
Vytla and Gebauer (2017)	Australian Dental Journal (Q3)	Odontogenic infections	NR	NR	Open access	Yes
Wright et al. (2016)	Journal of the ADA (Q2)	Cariology	AGREE	Industry, research, and institutional funds disclosed	Open access	Yes

AAPD, American Academy of Pediatric Dentistry; ADA, American Dental Association; AGREE, Appraisal of Guidelines, Research, and Evaluation; EFP, European Federation of Periodontology; IF, Impact Factor; Q, quartile; NA, not applicable.

### Adherence to the AGREE Reporting Guideline

The mean total adherence among the sample was 48.7%, ranging in the item analysis from 100% to 2.7% (Table 2 and Figure). Overall, the mean adherence varied across items (Table 2). The “Objectives” (#1) item was the only one with a complete adherence rate in all guidelines (100%, *n* = 21). The lowest reported item was “Implementation Advice/Tools” (#19), with an adherence rate of 3.2% (95% CI: 0.3%–6.0%). On the domain level (Table 3), the “Clarity of Presentation” item collected the highest average adherence rate (83.6%, 95% CI: 75.4%–91.8%), while the “Applicability” domain had the lowest (18.5%, 95% CI: 11.4%–25.6%).

**Table 2.** Mean Adherence across Each Item of the AGREE Checklist in Oral Health Clinical Guidelines (*n* = 21).^a^

AGREE Item	Mean Adherence (%)	Overall Completeness (%)
1. Objectives	100.0 (100.0 to 100.0)	100.0 (100.0 to 100.0)
2. Questions	79.0 (70.7 to 87.4)	38.1 (13.3 to 58.9)
3. Population	53.3 (42.1 to 64.6)	4.8 (−4.3 to 13.9)
4. Group Membership	64.8 (50.2 to 79.3)	23.8 (5.6 to 42.0)
5. Target Population Preferences and Views	21.4 (5.1 to 37.7)	14.3 (−0.7 to 29.3)
6. Target Users	95.2 (85.9 to 104.6)	95.2 (86.1 to 104.3)
7. Search Methods	35.7 (23.7 to 47.7)	4.8 (−4.3 to 13.9)
8. Evidence Selection Criteria	31.0 (16.5 to 45.4)	4.8 (−4.3 to 13.9)
9. Strengths and Limitations of the Evidence	67.3 (50.7 to 84)	38.1 (13.3 to 58.9)
10. Formulation of Recommendations	39.7 (21.9 to 57.5)	23.8 (5.6 to 42.0)
11. Consideration of Benefits and Harms	79.8 (65.6 to 94)	66.7 (46.5 to 86.8)
12. Link between Recommendations and Evidence	49.2 (29.8 to 68.7)	38.1 (13.3 to 58.9)
13. External Review	22.9 (9.3 to 36.5)	0.0 (0.0 to 0.0)
14. Updating Procedure	36.5 (19.7 to 53.3)	14.3 (−0.7 to 29.3)
15. Specific and Unambiguous Recommendations	87.6 (80.7 to 94.5)	57.1 (36.0 to 78.3)
16. Management Options	90.5 (79.5 to 101.4)	85.7 (70.7 to 100.7)
17. Identifiable Key Recommendations	66.7 (47.1 to 86.2)	61.9 (41.1 to 82.7)
18. Facilitators and Barriers to Application	40.5 (26.3 to 54.6)	4.8 (−4.3 to 13.9)
19. Implementation Advice/Tools	3.2 (0.3 to 6.0)	0.0 (0.0 to 0.0)
20. Resource Implications	25.0 (11.5 to 38.5)	4.8 (−4.3 to 13.9)
21. Monitoring/Auditing Criteria	13.1 (0.2 to 26)	4.8 (−4.3 to 13.9)
22. Funding Body	35.7 (21.9 to 49.5)	9.5 (−3.0 to 22.1)
23. Competing Interests	36.9 (20.5 to 53.3)	9.5 (−3.0 to 22.1)
Total	48.7 (40.2 to 57.1)	29.7 (10.2 to 49.2)

AGREE, Appraisal of Guidelines, Research, and Evaluation.

a Data are presented as the mean and 95% confidence interval (CI).

**Figure.**
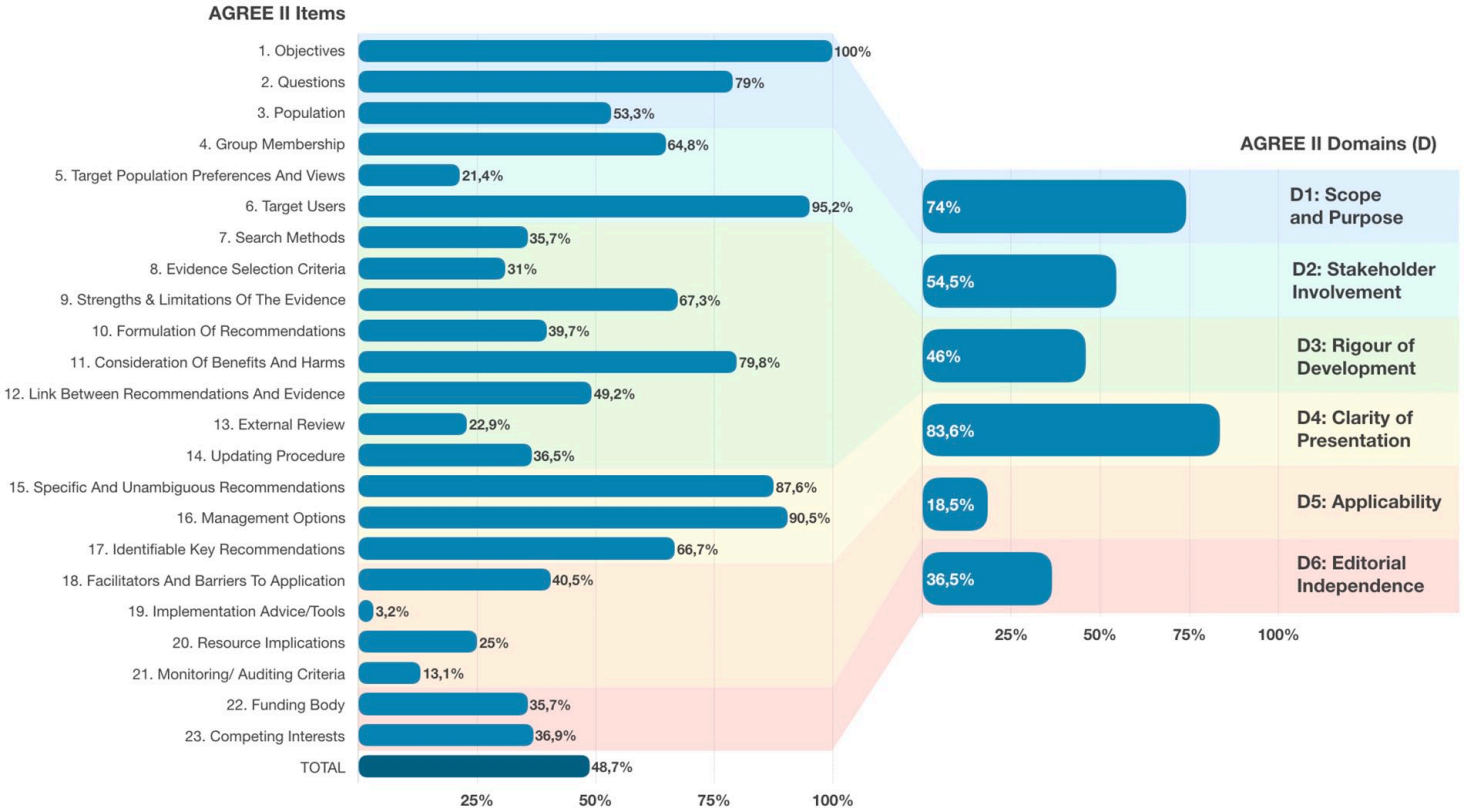
Adherence across AGREE items, domains, and total adherence. From left to right: the image aggregates each item into each domain via colorimetric division. In the bottom of the left graph, total adherence appears in a dark blue bar. AGREE, Appraisal of Guidelines, Research, and Evaluation.

**Table 3.** Mean Adherence across Each Domain of the AGREE Checklist in Oral Health Clinical Guidelines (*n* = 21).

AGREE Domain	Mean Adherence (%)	Overall Completeness (%)
Domain 1: Scope and Purpose	74.0 (67.1–80.9)	0.0 (0.0–0.0)
Domain 2: Stakeholder Involvement	54.5 (43.4–65.7)	4.8 (0.0–22.7)
Domain 3: Rigor of Development	46.0 (33.9–58.1)	0.0 (0.0–0.0)
Domain 4: Clarity of Presentation	83.6 (75.4–91.8)	42.9 (24.5–63.5)
Domain 5: Applicability	18.5 (11.4–25.6)	0.0 (0.0–0.0)
Domain 6: Editorial Independence	36.5 (22.5–50.5)	0.0 (0.0–0.0)
Mean	52.2 (44.1–60.3)	7.9

AGREE, Appraisal of Guidelines, Research, and Evaluation.

The linear regression analysis showed a significant relationship between journal quartile and guideline endorsement by the journal but not with publication year (Table 4). Publishing options were not included in the end because all guidelines were published as open access. In the adjusted model (model 2), guidelines published in the second- (B = −27.3%; SE = 6.1, *P* = 0.0002) and third-quartile (B = −22.8%; SE = 10.6, *P* = 0.0435) journals and in journals that have not endorsed a priori reporting guidelines (B = −20.9%, SE = 5.9, *P* = 0.0027) displayed a lower overall completeness than those published in first-quartile journals. In an item-wise analysis (Appendix Table 4), the adjusted model showed third-quartile journals (B = −20.3%; SE = 7.3, *P* = 0.0137) and journals that have not endorsed a priori reporting guidelines (B = −16.7%, SE = 5.4, *P* = 0.0067) displayed lower item completeness.

**Table 4.** Multivariable Linear Regression Models for the Association between Independent Variables and Overall Completeness to the AGREE Checklist.^a^

	B (95% CI)	Standard Error B	*P* Value
Model 1 (*R*^ 2^ = 0.717)			
Constant	−1,015.1 (−6,418.7; 4,388.5)	2,535.2	0.6945
Quartile (Q1 vs. Q2)	−21.8 (−34.8; −8.8)	6.1	0.0027
Quartile (Q1 vs. Q3)	−25.7 (−44.5; −6.8)	8.8	0.0101
Year	0.5 (−2.1; 3.2)	1.3	0.6756
Guidelines not endorsed by the journal	−20.7 (−33.7; −7.8)	6.1	0.0039
Model 2 (*R*^ 2^ = 0.731)			
Constant	66.1 (58.8;73.5)	3.5	<0.0001
Quartile (Q1 vs. Q2)	−22.0 (−34.5; −9.4)	5.9	0.0019
Quartile (Q1 vs. Q3)	−27.0 (−44.1; −9.9)	8.1	0.0041
Guidelines not endorsed by the journal	−20.9 (−33.5; −8.4)	5.9	0.0027

AGREE, Appraisal of Guidelines, Research, and Evaluation; B, unstandardized Beta coefficient; CI, confidence interval; Q1, quartile 1; Q2, quartile 2; Q3, quartile 3.

a The dependent variable is the mean overall adherence to the AGREE checklist.

## Discussion

This study examined the extent to which oral and dental CPGs comprehensively adhere to CPG reporting guidelines and assessed the existing links with editorial and study features. The study’s findings indicated that the level of reporting completeness was low. Specifically, the overall conformity to the AGREE checklist was 48.7%, ranging from 14.1% to 76.1% (as detailed in Appendix Table 3).

These results are consistent with those reported from CPGs published until 2014, as the adherence to reporting guidelines was suboptimal (Mubeen et al. 2017) with an average adherence of 51.9%. In this study, the authors reported that the quality of the guidelines improved per publication year, yet no such association was observed in the present study. Surprisingly, despite the warning elicited by this study, the quality of compliance of CPGs remains inadequate.

Improvement is necessary for all stakeholders involved in the evidence production process, yet CPG developers must enhance compliance with reporting standards and increase the readability and transparency of their research reports. Future developers of CPGs are expected to adhere to the reporting guidelines and provide supplementary materials, including appendices and raw data, in sufficient depth to allow for the assessment of their work by peers and readers, and to facilitate the application of their findings. Yet, in the context of dental and oral health, the results suggest that reporting completeness tends to be higher in journals that endorse reporting guidelines, such as AGREE, during the submission process. This suggests that adherence to reporting guidelines not only enhances the transparency and reproducibility of research findings but also reflects the methodological rigor and quality of the studies themselves. Therefore, it is possible that reverse causality is at play in this matter, as it appears that authors report better because top journals endorse reporting guidelines, rather than the other way around, where authors publish in top journals because they report better. For this reason, the findings highlight the pivotal role played by journals and editors in increasing adherence to CPG reporting guidelines and the need for all journals to endorse it to change the “reporting” culture.

While this study primarily focused on analyzing the adherence to CPGs, it is pertinent to discuss why all the identified CPGs were published as open-access articles. The trend of open access in scientific information dissemination is gaining acceptance among researchers, funding organizations, and publishers owing to its ability to promote transparency, accessibility, and equity. The open-access model supports evidence-based practice by providing guidelines free of charge to health care professionals, researchers, policymakers, and the general public, removing barriers (e.g., subscription fees or paywalls). Funding bodies and institutions mandating open-access publishing, along with the academic community’s growing support, may have encouraged authors and publishers to choose open-access dissemination for CPGs. In addition, the digital age has made open-access publishing platforms more widely available, enabling authors to reach a global audience and increase the influence of their guidelines.

The role of CPGs in health care is key as they provide evidence-based recommendations to guide clinical decision-making and improve patient outcomes. They are developed through a rigorous process that involves systematically reviewing the available evidence, synthesizing it, and translating it into actionable recommendations for health care practitioners (Brouwers et al. 2016). The importance of CPGs lies in their ability to standardize and optimize clinical care, enhance patient safety, and promote efficiency within healthcare systems. By providing clear guidance based on the best available evidence, CPGs help clinicians make informed decisions, reduce unwarranted variations in practice, and ultimately improve the quality of patient care (AGREE Collaboration 2003; Brouwers et al. 2010; Institute of Medicine Committee on Standards for Developing Trustworthy Clinical Practice Guidelines et al. 2011).

In addition to the methodological biases inherent in incomplete adherence to CPG guidelines, such as bias and conflicts of interest, incomplete or inaccurate information, or difficulty in replication and validation, there may be additional risks that are more hazardous. Failure to fully comply with reporting guidelines could result in methodological shortcomings that may pose risks to patients by advocating for interventions that are ineffective, unneeded, or even detrimental. Guidelines serve a crucial role in facilitating informed treatment decisions for both patients and health care providers. However, when guidelines lack transparency, it can result in suboptimal care and undesirable consequences. Also, endorsing the use of reporting guidelines in dentistry often results in better reporting (Sarkis-Onofre et al. 2020).

Furthermore, the insufficient participation of stakeholders found in this study, especially patients, in the formulation of CPGs that do not adequately meet the requirements and preferences of their intended users may reduce their significance and practicality in clinical practice. Stricter adherence to these guidelines could improve the quality and reliability of available clinical information, which is essential to inform decision-making in oral health. In addition, this study highlights the need for future research aimed at identifying specific barriers to adherence to reporting guidelines in clinical practice as well as developing effective strategies to overcome them. Improving compliance with reporting guidelines would lead to an improvement in the quality and impact of oral health research and, on a wider scale, improve treatment conditions for patients.

Overall, there is a need for future research to adopt enhanced methodological rigor and adhere to standardized reporting requirements to further improve the quality and transparency of CPGs in oral and dental health. Researchers developing CPGs should prioritize the integration of adequate reporting criteria to ensure that guidelines are comprehensive, methodologically sound, and user-friendly. Including clear methodologies for stakeholder involvement—especially patients, who bring essential perspectives to guideline development—would increase the relevance and practicality of CPGs in clinical practice. In addition, adopting more systematic approaches for documenting and addressing potential conflicts of interest is essential, as these factors significantly affect guideline integrity and reliability. Academic journals must bolster their commitment to and enforcement of CPG reporting standards. This action will ensure future guidelines address editorial autonomy and other crucial aspects with transparency, ultimately enhancing the credibility and impartiality of published guidelines. The standardization of these methodological elements across future CPGs could potentially enhance consistency, mitigate potential biases, and ultimately improve the clinical applicability and trustworthiness of guidelines within the field.

### Study Limitations

The evaluation of quality for our study involved the use of cross checks based on the AGREE criteria. Although the existence of alternative reporting guidelines is acknowledged, such as the Reporting Items for Practice Guidelines in Healthcare (RIGHT), both AGREE and RIGHT share 11 common items (Yao et al. 2020). It is recommended that developers of CPGs use either AGREE plus items unique to RIGHT or RIGHT plus items unique to AGREE (Yao et al. 2020). That being said, the AGREE Reporting Checklist has slight preference due to its origins in an internationally recognized standard for assessing the methodological quality of CPGs (e.g., AGREE). Lastly, the search was restricted to CPGs published from 2016 onward, given that the AGREE reporting guideline was released in that same year.

Another shortcoming is that CPGs were searched on journal databases and not in repositories such as GIN (https://g-i-n.net/) (Ollenschlager et al. 2004), and this might have influenced the overall validity of our findings. However, such databases may be less known to clinicians who are unaware of their existence and primarily rely on published articles as their reference for daily practice. Another limitation is the challenge of accessing certain unpublished or less accessible CPGs. This restricts the breadth of our analysis, as potentially relevant guidelines could not have been included for analysis (Wilkinson et al. 2017). In addition, certain CPGs may exist in proprietary or subscription-only databases, creating access barriers that limit comprehensive data collection (Shiffman 2009; Bierbaum et al. 2022).

Another shortcoming is the limitation regarding the number of assessors. While AGREE recommends evaluations by 4 reviewers to enhance reliability, our study employed 2 independent assessors who conducted evaluations separately, followed by a consensus process to resolve discrepancies. This approach, although not optimal, aligns with a previous agreement study (Jo et al. 2013) and ensures a systematic and transparent evaluation. We acknowledge that increasing the number of evaluators could further enhance reliability and that this is a study limitation. Also, AGREE is a tool for evaluating both reporting and methodological quality, but readers shall notice that in this article, it was used as a methodological adherence tool, focusing on the extent to which the included guidelines followed established development standards rather than solely assessing their reporting clarity.

## Conclusion

The level of completeness in dental/oral CPGs is suboptimal and linked to their journal’s quartile ranking and reporting guideline endorsement. This highlights the need for researchers, authors, and publishers to have a better understanding of the guidelines for reporting CPGs and to make more strenuous efforts to ensure their proper application. In addition, this study highlights the key role played by journals and editors in endorsing reporting guidelines as they are key allies in improving reporting in oral health research.

## Author Contributions

P. Lancry, contributed to conception, design, data acquisition, drafted and critically revised the manuscript; P. Lyra, J.J. Mendes, G.G. Nascimento, contributed to data interpretation, critically revised the manuscript; V. Machado, contributed to design, data interpretation, critically revised the manuscript; J. Botelho, contributed to conception, design, data acquisition, analysis, and interpretation, drafted and critically revised the manuscript. All authors gave final approval and agree to be accountable for all aspects of the work.

## Supplemental Material

sj-docx-1-jct-10.1177_23800844251357235 – Supplemental material for Evaluating Reporting Completeness in Oral Health Clinical Guidelines: A Meta-Research Study Supplemental material, sj-docx-1-jct-10.1177_23800844251357235 for Evaluating Reporting Completeness in Oral Health Clinical Guidelines: A Meta-Research Study by P. Lancry, P. Lyra, J.J. Mendes, G.G. Nascimento, V. Machado and J. Botelho in JDR Clinical & Translational Research

sj-xlsx-2-jct-10.1177_23800844251357235 – Supplemental material for Evaluating Reporting Completeness in Oral Health Clinical Guidelines: A Meta-Research Study Supplemental material, sj-xlsx-2-jct-10.1177_23800844251357235 for Evaluating Reporting Completeness in Oral Health Clinical Guidelines: A Meta-Research Study by P. Lancry, P. Lyra, J.J. Mendes, G.G. Nascimento, V. Machado and J. Botelho in JDR Clinical & Translational Research
