# Supplementary material for: Evaluating Reporting Completeness in Oral Health Clinical Guidelines: A Meta-Research Study
Source: JDR Clin Trans Res. 2025 Aug 16;11(3):412–22. doi: 10.1177/23800844251357235 (PMC13232573; doi:10.1177/23800844251357235)
Supplement: Supplementary file 1 [file sj-docx-1-jct-10.1177_23800844251357235.docx]

**Appendices**

**Evaluating Reporting Completeness in Oral Health Clinical Guidelines: a meta-epidemiologic study**

Pierre Lancry, Patrícia Lyra, José João Mendes, Gustavo G. Nascimento, Vanessa Machado, João Botelho

**Appendix Table 1. Guideline for reporting meta-epidemiological methodology research by Murad and Wang** (Murad and Wang 2017).

| **Section/Item** | **Description** | Page |
| --- | --- | --- |
| **Title** | Identify the report as a meta-epidemiologic study. | 1 |
| **ABSTRACT** |  |  |
| Structured summary | Provide a structured summary that includes the background of the topic, goal of the study, data sources, method of data selection, appraisal and synthesis methods, results, limitations, conclusions and implications of key findings. | 2 |
| **INTRODUCTION** |  |  |
| Rationale | Describe the rationale for the meta-epidemiological study in the context of what is already known. | 3 |
| Objectives | Provide an explicit statement of the goal of the meta-epidemiological study and the hypothesis being empirically tested. | 3 |
| **METHODS** |  |  |
| Protocol | Indicate if a protocol exists, if and where it can be accessed (eg, Web address). Registration of a protocol is not mandatory. | 4 |
| Eligibility criteria | Specify study characteristics used as criteria for eligibility with a rationale. | 4 |
| Information sources | Describe all information sources (eg, databases with dates of coverage, contact with experts to identify additional studies, Internet searches) and search date. | 4 |
| Search | Present full electronic search strategy for at least one database, including any limits used, such that it could be repeated. Search is commonly not driven by a clinical question. | 4 |
| Study selection | Describe the process for selecting studies for inclusion (ie, how many reviewers selected studies, reviewing in duplicate or by single individuals). | 4 |
| Data collection process | Describe method of data extraction from reports (eg, piloted forms, independently, in duplicate) and any processes used for manipulating data or obtaining and confirming data from investigators. | 4 |
| Data items | List and define all variables for which data were sought and any assumptions and imputations made. | 4 |
| Risk of bias in individual studies | If risk of bias assessment of individual studies was relevant to the analysis, describe the items used and how this information is to be used during data synthesis. | NA |
| Summary measures | State the principal summary measures (eg, ratio of risk ratios, difference in means) and explain its meaning and direction to readers. | 5 |
| Synthesis of results | Describe the statistical or descriptive methods of synthesis including measures of consistency if relevant. If applicable, describe the development of statistical or simulation modelling based on theoretical background. Describe and justify assumptions and computational approximations. Describe methods of additional analyses (eg, sensitivity or subgroup analyses, meta-regression), if done, indicating which were prespecified. | 5-6 |
| **RESULTS** |  |  |
| Study selection | Give numbers of studies assessed for eligibility and included in the study, with reasons for exclusions at each stage, ideally with a flow diagram. Present a measure of inter-reviewer agreement (eg, kappa statistic). | 7 |
| Study characteristics | For each study, present characteristics for which data were extracted and provide the citations. Clinical characteristics may not always be relevant. | 7 |
| Risk of bias within studies | If risk of bias assessment of individual studies was used in the meta-epidemiological analysis, report risk of bias indicators of each study to allow replication of findings. | 7 |
| Results of individual studies | Present data elements used in the meta-epidemiological analysis from each study (results of clinical outcomes may not be relevant). | 7 |
| Synthesis of results | Present results of statistical analysis done, including measures of precision and measures of consistency. Present validity of assumptions and fit of statistical or simulation modelling, if applicable. | 7 |
| Additional analysis | Give results of additional analyses, if done (eg, sensitivity or subgroup analyses, meta-regression). | 7 |
| **DISCUSSION** |  |  |
| Summary of evidence | Summarise the main findings and compare them with existing knowledge about the topic. The quality of evidence may not be relevant; however, investigators should describe their certainty in the results to readers. | 8-9 |
| Limitations | Discuss limitations at research methodology level (eg, likelihood of reporting or publication bias). | 9-10 |
| Conclusions | Provide general interpretation of the results and implications for future research. Provide any plausible impact on clinical practice. | 10 |
| **FUNDING** | Describe sources of funding for the methodology research and role of funders. | 11 |

Murad MH, Wang Z. 2017. Guidelines for reporting meta-epidemiological methodology research. Evid Based Med. 22(4):139–142.

**Appendix Table 2. Search syntaxes**

PUBMED

("Clinical Practice Guideline" OR "Practice Guideline") AND ("Dental Pain" OR "Pharmacologic Management" OR "Restorative Treatments" OR "Caries Lesions" OR "Peri-implant Diseases" OR Periodont* OR "Traumatic Dental" OR "Osteonecrosis of the Jaw" OR "COVID-19" OR "Antibiotics" OR "Pulp Therapy" OR "Fluoride" OR "Nonrestorative Treatments" OR "Potentially Malignant Disorders" OR "Primary Teeth" OR "Silver Diamine Fluoride" OR "Odontogenic Infections" OR "Sealants")

n = 349

WEB OF SCIENCE

("Clinical Practice Guideline" OR "Practice Guideline") AND ("Dental Pain" OR "Restorative Treatments" OR "Caries Lesions" OR "Peri-implant Diseases" OR Periodont* OR "Traumatic Dental" OR "Osteonecrosis of the Jaw" OR "Antibiotics" OR "Pulp Therapy" OR "Fluoride" OR "Nonrestorative Treatments" OR "Potentially Malignant Disorders" OR "Primary Teeth" OR "Silver Diamine Fluoride" OR "Odontogenic Infections" OR "Sealants")

n = 546

EMBASE

('clinical practice guideline'/de OR 'practice guideline'/de) AND ('dental pain'/de OR 'restorative treatments' OR 'caries lesions' OR 'peri-implant diseases' OR periodont* OR 'traumatic dental' OR 'osteonecrosis of the jaw'/de OR 'antibiotics'/de OR 'pulp therapy' OR 'fluoride'/de OR 'nonrestorative treatments' OR 'potentially malignant disorders' OR 'primary teeth'/de OR 'silver diamine fluoride'/de OR 'odontogenic infections' OR 'sealants') AND [2016-2023]/py

n = 9,568

**Appendix Table 3. Excluded studies with reasons**

| **Study** | **Reason** |
| --- | --- |
| Sollecito TP, Abt E, Lockhart PB, Truelove E, Paumier TM, Tracy SL, Tampi M, Beltrán-Aguilar ED, Frantsve-Hawley J. The use of prophylactic antibiotics prior to dental procedures in patients with prosthetic joints: Evidence-based clinical practice guideline for dental practitioners--a report of the American Dental Association Council on Scientific Affairs. J Am Dent Assoc. 2015 Jan;146(1):11-16.e8. doi: 10.1016/j.adaj.2014.11.012. Epub 2014 Dec 18. PMID: 25569493. | Before 2016 (year of the AGREE publication) |
| Smiley CJ, Tracy SL, Abt E, Michalowicz BS, John MT, Gunsolley J, Cobb CM, Rossmann J, Harrel SK, Forrest JL, Hujoel PP, Noraian KW, Greenwell H, Frantsve-Hawley J, Estrich C, Hanson N. Evidence-based clinical practice guideline on the nonsurgical treatment of chronic periodontitis by means of scaling and root planing with or without adjuncts. J Am Dent Assoc. 2015 Jul;146(7):525-35. doi: 10.1016/j.adaj.2015.01.026. PMID: 26113100. | Before 2016 (year of the AGREE publication) |
| Wilson A, Hoang H, Bridgman H, Crocombe L, Bettiol S. Clinical practice guidelines and consensus statements for antenatal oral healthcare: An assessment of their methodological quality and content of recommendations. PLoS One. 2022 Feb 3;17(2):e0263444. doi: 10.1371/journal.pone.0263444. PMID: 35113944; PMCID: PMC8812839. | Unsuitable methodology |
| Oral Health Division, Ministry of Health Malaysia. Antibiotic Prophylaxis in Oral Surgery for Prevention of Surgical Site Infection (2nd Edition). ISBN: 9789670769394 | Before 2016 (year of the AGREE publication) |
| Levin L, Day PF, Hicks L, O'Connell A, Fouad AF, Bourguignon C, Abbott PV. International Association of Dental Traumatology guidelines for the management of traumatic dental injuries: General introduction. Dent Traumatol. 2020 Aug;36(4):309-313. doi: 10.1111/edt.12574. Epub 2020 Jun 22. PMID: 32472740. | Not a guideline |
| AAE Position Statement: AAE Guidance on the Use of Systemic Antibiotics in Endodontics. J Endod. 2017 Sep;43(9):1409-1413. doi: 10.1016/j.joen.2017.08.015. PMID: 28844223. | Not a guideline |
| Bidra AS, Daubert DM, Garcia LT, Kosinski TF, Nenn CA, Olsen JA, Platt JA, Wingrove SS, Chandler ND, Curtis DA. Clinical practice guidelines for recall and maintenance of patients with tooth-borne and implant-borne dental restorations. Gen Dent. 2016 Jan-Feb;64(1):14-21. PMID: 26742161. | Unable to access the methodology |
| Oh TJ, Yu SH. Evidence-based clinical practice guideline for treatment of stage IIII periodontitis. J Evid Based Dent Pract. 2021 Dec;21(4):101638. doi: 10.1016/j.jebdp.2021.101638. Epub 2021 Aug 30. PMID: 34922721. | Commentary |
| Coté CJ, Wilson S; AMERICAN ACADEMY OF PEDIATRICS; AMERICAN ACADEMY OF PEDIATRIC DENTISTRY. Guidelines for Monitoring and Management of Pediatric Patients Before, During, and After Sedation for Diagnostic and Therapeutic Procedures. Pediatrics. 2019 Jun;143(6):e20191000. doi: 10.1542/peds.2019-1000. PMID: 31138666. | Unrelated |
| Diogenes, Christine M. Sedgley, and Bruce Y. Cha. "AAE Guidance on antibiotic prophylaxis for patients at risk of systemic disease." American Association of Endodontists (2017): 1-6. | Not a guideline |
| Troubles temporo-mandibulaires: lignes directrices et soins personnels pour les patients pendant la pandémie de COVID-19 | Not a guideline |

**Appendix Table 4. Multivariable linear regression models for the association between independent variables and item completeness to the AGREE Checklist.**

|  | B (95 % CI) | Std. Error B | P-value |
| --- | --- | --- | --- |
| Model 1 (R^2^= 0.549) |  |  |  |
| Constant | -1915.6 (-6745.3;2914.1) | 2265.9 | 0.4112 |
| Quartile (Q1 vs. Q2) | -9.9 (-21.5;1.7) | 5.4 | 0.0898 |
| Quartile (Q1 vs. Q3) | -17.9 (-34.7;-1.1) | 7.9 | 0.0385 |
| Year | 0.9 (-1.4;3.4) | 8.6 | 0.4012 |
| Guidelines not endorsed by the Journal | -16.4 (-28.0;-4.8) | 5.4 | 0.0088 |
| Model 2 (R^2^= 0.556) |  |  |  |
| Constant | 42.0 (35.4;48.7) | 3.2 | <0.0001 |
| Quartile (Q1 vs. Q2) | -10.2 (-21.6;1.2) | 5.4 | 0.0759 |
| Quartile (Q1 vs. Q3) | -20.3 (-35.9;-4.8) | 7.3 | 0.0137 |
| Guidelines not endorsed by the Journal | -16.7 (-28.1;-5.3) | 5.4 | 0.0067 |

Note: Dependent variable is mean overall adherence to the AGREE checklist. Abbreviations: B - unstandardized Beta coefficient; CI – Confidence Interval; Q1 - Quartile 1; Q2 - Quartile 2; Q3 - Quartile 3.

**Appendix Figure 1. Flowchart of studies**
